# Exploring barriers to the effective use of computer-based simulation in pharmacy education: a mixed-methods case study

**DOI:** 10.3389/fmed.2024.1448893

**Published:** 2024-08-29

**Authors:** Ahmed M. Gharib, Gregory M. Peterson, Ivan K. Bindoff, Mohammed S. Salahudeen

**Affiliations:** School of Pharmacy and Pharmacology, College of Health and Medicine, University of Tasmania, Hobart, TAS, Australia

**Keywords:** computer-based simulation, pharmacy education, educational technology, interprofessional learning, curriculum integration, simulation-based learning, mixed-methods, virtual patients

## Abstract

**Background:**

At the University of Tasmania (UTAS), pharmacy education traditionally relies on placements to provide students with hands-on experience. However, these placements have become increasingly limited due to logistical challenges and growing student numbers. Computer-based simulation (CBS) has the potential to offer a scalable, effective alternative to enhance learning and critical thinking. However, integrating CBS in pharmacy education faces several barriers that must be addressed for successful implementation.

**Objective:**

To gain insight into pharmacy educators’ and students’ views regarding the barriers, and potential solutions, to integrating CBS in pharmacy practice education.

**Methods:**

This mixed-methods case study involved semi-structured interviews with pharmacy educators and quantitative surveys with pharmacy students. The data underwent thematic coding for interview transcripts and statistical analysis for survey responses. The findings were integrated by examining convergence, complementarity, and discrepancy, revealing insights into how pharmacy students and educators perceive implementation barriers and improvement strategies for CBS.

**Results:**

Ten interviews were conducted, and 75 survey responses were collected, with a 62.5% response rate. Key barriers to CBS integration included educators’ heavy workload, scepticism about CBS’s educational value, and general integration challenges. Students, however, showed high acceptance of CBS, with 70.7% agreeing that CBS could assess their knowledge, 69.3% emphasising its role in developing problem-solving skills, and 80% viewing CBS as a complement to classroom study. Proposed solutions for enhancing CBS uptake included additional institutional support by appointing dedicated simulation technicians, leveraging champions to advocate for CBS, and aligning CBS with educational objectives.

**Conclusion:**

A significant gap between students’ readiness and educators’ hesitancy to use CBS in pharmacy education was identified. While students are eager to adopt new technologies, educators expressed reservations, primarily due to workload concerns and uncertainties about the efficacy of CBS. The feedback from educators suggests that institutions may see improved uptake by employing dedicated support personnel and initiating targeted training programs. Future research should focus on exploring barriers and facilitators, using larger and more diverse samples, and gaining deeper insights into decision-makers’ perspectives to enhance the integration and efficacy of CBS in pharmacy education.

## 1 Introduction

In pharmacy education, students typically participate in workplace-integrated learning placements, which immerse them in supervised, real-world pharmacy practice scenarios. Although the educational value of these placements is widely recognised (1, 2), their availability is constrained by an increase in student numbers and the rising difficulties and costs associated with facilitating such experiences, leading to challenges in offering enough high-quality clinical placements (3). With reduced placement opportunities, increased emphasis on preparatory exercises seems prudent, but traditional classroom exercises such as paper-based cases and standardised patients (role-play) have a significant cost associated with marking and delivery on a per-student basis, which impacts the ability to scale up. This predicament underscores the importance of integrating complementary training methods, like computer-supported learning, which has demonstrated significant advantages in knowledge acquisition through its interactive and engaging approach, and which can be scaled up without a significant increase in delivery cost per student (4). Computer-supported learning aligns with Kolb’s experiential learning cycle by simulating real-life challenges in a safe environment, thus enhancing students’ critical thinking and collaborative skills, which are essential for their professional competency (1, 5).

At the University of Tasmania (UTAS), the demand for quality workplace-integrated learning placements in pharmacy is intensifying, exacerbated by growing student populations and logistical challenges across diverse campus locations. These issues pose a risk to maintaining educational standards and preparing a competent healthcare workforce, potentially impacting patient safety (2, 6). Innovative new educational tools such as computer-based simulation (CBS) and virtual patient training, present promising solutions that aim to enhance teaching and learning experiences by making them more engaging, efficient, and accessible, thereby enriching experiential learning opportunities and bridging the gap between theoretical knowledge and practical skills (4, 7–9). CBS, in particular, has been identified as a key tool in advancing pharmacy students’ competencies in critical areas where hands-on experience may be limited (10, 11).

Despite the local development of ‘Pharmacy Simulator’ by the School of Pharmacy and Pharmacology at UTAS (12), which was designed to be used across several subjects within the pharmacy curriculum, including pharmacotherapy, clinical pharmacy, and pharmaceutical care. The simulator is designed to replicate various pharmacy practice scenarios, allowing students to apply theoretical knowledge in a controlled, virtual environment. It enables students to practice decision-making, patient counselling, and medication management in both community and hospital pharmacy settings, without the risks associated with real-life errors (13, 14). However, there is limited engagement with this simulation tool among educators. This reflects a broader trend where advancements like CBS are not being fully integrated into pharmacy education, especially compared to other health disciplines (13, 14). As a result, this study investigated the perceptions of pharmacy educators and students at UTAS regarding the barriers to integrating CBS into pharmacy practice education.

## 2 Methods

A mixed-methods case-study design was selected for its ability to provide in-depth contextual insights and actively engage stakeholders, which is crucial for understanding the intricate dynamics between technology, pedagogy, and institutional culture (15, 16). This case study aimed to address any potential challenges to the uptake of ‘Pharmacy Simulator’ and other CBS systems at the School of Pharmacy and Pharmacology, University of Tasmania, Australia. ‘Pharmacy Simulator’ is an in-house simulator specifically designed to enhance pharmacy education by providing a realistic and interactive learning environment (12). This CBS allows students to engage in simulated clinical scenarios, fostering critical thinking and soft skills essential for pharmacy practice.

The study involved surveys and interviews conducted between March to September 2023 to gather diverse stakeholder perspectives. Surveys were administered to students, while interviews were conducted with educators. The mixed-methods approach allowed for a comprehensive understanding of both quantitative and qualitative data, providing a holistic view of the perceptions of CBS. Given the small population size, we employed a total population sampling approach, inviting all members of the study population to participate.

All UTAS pharmacy students were eligible to participate. A link to the online survey was distributed via emails, and the institution’s learning management system. Participation was voluntary, with incentives including a chance to win one of ten $50 AUD gift vouchers. Implied consent was obtained at the survey’s start. The survey included questions designed to assess students’ opinions on the use of CBS technology, perceived implementation barriers, and their general interest in the adoption of this technology within the curriculum. The online survey was developed and administered using LimeSurvey^®^ (LimeSurvey GmbH, Hamburg, Germany. URL http://www.limesurvey.org).

The initial constructs of the survey questions and overall design were identified based on a relevant literature search (13, 14) and scoping team meetings. Items were drafted and finalised using an iterative approach. The draft survey underwent independent content validation by two postgraduate pharmacy students and two pharmacy educators. Additionally, the survey was pre-tested with a small group of volunteers to assess its clarity and relevance, further supporting its face validity. These steps ensured that the survey was both comprehensive and aligned with the study’s objectives. The survey consisted of 20 questions, primarily close-ended for quantitative analysis, along with a 6-point Likert scale to measure respondents’ attitudes. The data were extracted from LimeSurvey^®^ to IBM Statistical Package for Social Sciences (IBM SPSS^®^ Statistics for Windows, version 26.0; IBM Corp, Armonk, NY, United States). Descriptive analysis was primarily used for the quantitative part of the study. Chi-square test was applied to make comparisons between groups, and a *p*-value <0.05 was considered statistically significant.

### 2.1 Design of the qualitative phase—educators interview

An email invitation, which included a participant information sheet and consent form, was sent to all pharmacy practice educators (*n* = 16) to participate in a semi-structured interview. These interviews aimed to examine their perspectives on CBS, discussing both the opportunities and challenges associated with its practical implementation. The interviews also explored educators’ views on possible approaches to enhance the integration and use of CBS at the institution. An online 30-min interview was conducted with each educator who agreed to participate and provided written consent. The semi-structured interviews were conducted using an open-ended script to explore the participants’ views on CBS and experiences with existing models. The interview format employed a ‘funnel-shaped’ approach, narrowing down from broad to specific inquiries (17). The script was developed based on a literature review (13, 14) and team discussions, and was pre-tested with three educators. The interview guide was designed to address several key constructs, including general perceptions of CBS, the adequacy of institutional and educator support, student engagement with CBS, and strategies for optimizing CBS implementation. Specific questions included, for instance, “What are pharmacy educators’ views on incorporating CBS into pharmacy practice education?” and “Are pharmacy educators adequately skilled and willing to integrate CBS into curricula?”. The full interview guide is available in Appendix B.

At the completion of each interview, the audio recording was transcribed using Zoom (18), and de-identified by assigning unique codes to remove personal identifiers. The interview transcripts were analysed thematically following Braun and Clarke’s (19) approach. The coding process, guided by an initial codebook, was manually conducted using NVivo (version 12.4, 2020, QSR) (20). This iterative process combined inductive and deductive strategies, allowing themes to emerge naturally while applying theoretical frameworks for depth. Researchers (AG, MS) conducted the coding, engaging in regular discussions to refine the codebook and resolve discrepancies through consensus, thereby enhancing thematic reliability. Contextual reflexivity was used to critically assess how the educational environment at UTAS and the researchers’ positionality influenced the findings on CBS integration barriers. To mitigate potential biases, independent researchers cross-validated the themes, and an audit trail of analytical decisions was maintained to ensure the trustworthiness of the findings.

Ethical approval was granted from the University of Tasmania’s (UTAS) Human Research Ethics Committee (Project ID: 26897).

## 3 Results

### 3.1 Student survey

The survey had a response rate of 62.5%, with 75 out of 120 enrolled students participating. The participants represented different study years of the Bachelor of Pharmacy program, as seen in Supplementary Table S1. The survey data highlighted students’ access to technology resources, their positive perspectives on the integration of CBS in the curriculum, the importance of a user-friendly and engaging CBS design, and their training preferences. However, there were concerns about the expected support levels from educators and institution for CBS implementation.

Regarding the role of CBS in curriculum, a significant proportion agreed that CBS could be utilised for assessing their knowledge (70.7%), in the classroom (62.7%), and as a supplement to classroom study (80%). The students expressed strong interest in developing patient communication and counselling skills (68%), problem-solving skills (69.3%), and dispensing procedures (66.7%) through CBS (as seen in Table 1). These preferences align with the practical and interpersonal competencies required in the pharmacy profession, indicating that students recognised the potential of CBS in enhancing these essential skills.

**Table 1:** Students’ Perspectives on CBS success factors and training preferences (*N* = 75).

Themes	Description	CBS features	Very important, *N* (%)	Somewhat important, *N* (%)	Not important at all, *N* (%)	No opinion, *N* (%)
Important elements for successful CBS tool	Considering personal preference for elements that are crucial for CBS success	Realistic graphics	32 (42.7%)	29 (38.7%)	10 (13.3%)	4 (5.3%)
		Ease of use	60 (80%)	15 (20%)	–	–
		Bug-free experience	60 (80%)	15 (20%)	–	–
		Availability of technical support	36 (48%)	39 (52%)	–	–
		Detailed feedback	46 (61.3%)	22 (29.3%)	5 (6.7%)	2 (2.7%)
		Engaging content	52 (69.3%)	18 (24%)	3 (4%)	2 (2.7%)
		Fun/enjoyable content	43 (57.3%)	25 (33.3%)	4 (5.3%)	3 (4%)
Training preferences from a CBS tool	Considering personal views on the most suitable training focus for integrating CBS	Patient communication and counselling skills	51 (68%)	12 (16%)	8 (10.7%)	4 (5.3%)
		Problem-solving skills	52 (69.3%)	21 (28%)	1 (1.3%)	1 (1.3%)
		Dispensing procedures	50 (66.7%)	20 (26.7%)	3 (4%)	2 (2.7%)
		Interprofessional communication skills	36 (48%)	28 (37.3%)	2 (2.7%)	9 (12%)
		Hospital pharmacy practice	44 (58.7%)	26 (34.7%)	–	4 (5.3%)
		Community pharmacy practice	44 (58.7%)	22 (29.3%)	2 (2.7%)	7 (9.3%)
		Community-based clinic	30 (40%)	28 (37.3%)	2 (2.7%)	15 (20%)

CBS, computer-based simulation.

Table 2 shows students’ views on perceived support for CBS integration from different stakeholder groups. A chi-square test (*p*-value = 0.0002) confirmed significant differences in these perceptions. Notably, 37.3% of students believed their peers were very likely to support CBS, and 52% considered it somewhat likely. In contrast, only 16% perceived strong support from educators, with 33.3% seeing it as unlikely. Institutional support was also viewed critically, with only 20% seeing strong support and 40% considering it somewhat likely. These results highlight a gap in perceived support from educators and institutions compared to their peers.

**Table 2:** Students’ views on the expected support level for CBS integration from different stakeholder groups.

Stakeholders	Expected support level for CBS integration			*p*-value
	Very likely, *N* (%)	Somewhat likely, *N* (%)	Not likely, *N* (%)	
Students	28 (37.3%)	39 (52%)	8 (10%)	0.0002
Educators	12 (16%)	38 (50.7%)	25 (33.3%)	
Institution	15 (20%)	30 (40%)	30 (40%)	

Chi-square was applied.*p* < 0.05 is considered statistically significant, highlighted in bold font.CBS, computer-based simulation.

### 3.2 Educator interviews

Of the 16 invited, 10 pharmacy practice educators were interviewed, achieving saturation-level with no new themes or insights emerging after the tenth interview. The educators had diverse pharmacy practice teaching experience, academic roles and exposure to CBS, as summarised in Supplementary Table S3. Regarding their experience with CBS, only two of the interviewees were actively using CBS in their teaching at the time of the interview. Additionally, eight educators had previous exposure to CBS, having trialled or used it in the past, but were not actively using it currently and were not necessarily updated on the changes that have occurred in CBS design features (some had not used CBS for several years). Data saturation was achieved after conducting 10 interviews, as no new themes emerged. The analysis identified three main themes: resistance to change, implementation and operational challenges, and CBS alignment with the curriculum. Additionally, several subthemes emerged within these broader categories, each highlighting specific aspects of the educators’ experiences and concerns. The emerging themes and insights are described in Tables 3, 4.

**Table 3:** Selected quotes on barriers themes from educators’ interviews.

Sub-theme and responses (*n*)	Representative quotes
Theme 1—resistance to change	
Educators’ resistance to change Number of participants = 8 Sub-theme frequency = 11	*“I believe in the tried and tested. These tech solutions seem to complicate our traditional teaching methods without proven benefits.”*—*P2* *“…organisational and educator’s mindset is often difficult to change. Some people’s perceptions of it as a game that does not have educational merit and do not want to engage with it.”—P3* *“… some would never implement it because, you know, they do not like technology at all”—P10*
Perceived Student Resistance to Change Number of participants = 5 Sub-theme frequency = 12	*“For some students, it is just like, ‘No, that is not how I study. Especially, our international students, if told, ‘You are going to play a simulation game now,’ might misconstrue this as trivial rather than educational.”—P1* *“I think they want authentic experiences. I think they want the real-life thing and if they are given a computer simulation instead of the real-life thing, they will see it as a cheap cop-out.”*—*P8*
Theme 2—Implementation/operational challenges	
Educators’ workload and time Number of participants = 10 Sub-theme frequency = 32	*“It is very much about you design the whole content yourself and I know one of them used to take two to four days to develop a 15-min interaction, it’s a big limiting factor.”—P2* *“… this is not an easy thing, and it is very time-consuming. Everyone is extremely busy developing their courses.”—P3* *“Creating those cases, testing them out, probably it will take a couple of years to perfect the cases … we have a lot of increased workloads within the first couple of years, but it should be very minimal work from then on, once you have got a good case and it’s going well.”—P9*
Resource Constraints Number of participants = 5 Sub-theme frequency = 7	*“I question if we have the correct computers that can run the sim, especially with the graphics cards that are needed. There would be a financial implication if there was not sufficient IT support up there.”—P1* *“Accessing the simulations was quite the challenge. Slow devices often required the students to use their own devices, which led to compatibility and optimisation issues.”—P4*
Theme 3—CBS alignment with the curriculum	
Appropriateness of using CBS Number of participants = 7 Sub-theme frequency = 14	*“Just having a good time (for students), but absolutely achieving no educational outcomes.”*—*P1* *“It has its place, but the amount of evidence required to implement it is quite substantial.”*—*P8* *“Obviously, some educators do not see how it would fit in their units. So, we have got to overcome that kind of barrier before can use it.”*—*P10*
CBS design limitation Number of participants = 7 Sub-theme frequency = 12	*“If you are trying to teach complex communication skills, there are limitations to what the current CBS programs can offer. How do you, in a computer simulator, let a student know that they need to speak louder if the person’s an older person but then do not start patronising them, like talking to them like they are a little child—and use a louder voice, but same language.”—P2* *“I appreciate the potential of simulations for hands-on learning, but the one-size-fits-all approach does not account for different learning speeds and styles. A more tailored experience would help.”—P4*

CBS, computer-based simulation.

**Table 4:** Selected quotes on proposed solutions themes and its linkage to the corresponding barriers from educators’ interviews.

Sub-theme and responses (*n*)	Representative quotes
Solution 1—communicating CBS benefits	
Complementing placement training Number of participants = 10 Theme frequency = 18 Linked to barrier group: “CBS alignment within the pharmacy curriculum”	*“Even before placements occur, this could give students that experience of how to deal with real-world people before they actually begin to deal with real-world people.”—P5* *“I think the real-time feedback, being non-location based, non-time based, is the biggest value you get from these simulations compared to the lengthy coordination required to secure clinical placements.”—P8* *“Going into the hospital for teaching students there is very useful, but human resource timewise, it is very costly. A lot of these types of cases could be incorporated into a computer simulation to complement my students’ training.”—P9* *“You do not always get that (talk to patients) because they might be in isolation, grumpy, have visitors, going for a scope, or asleep. In computer simulation, cases are ready anytime, anywhere! The training quality may not be similar, but it is something better than nothing.”—P10*
Interprofessional training Number of participants = 9 Theme frequency = 15 Linked to barrier group: “CBS alignment within the pharmacy curriculum”	*“CBS can simulate a hospital situation, helping students understand what to ask a nurse or a doctor, enhancing IPL, especially when we are limited by how much IPL we can do with other disciplines because of timetabling and those sorts of things, you could see a sim environment for that being quite beneficial, particularly in a hospital situation.”—P1* *“I believe that incorporating computer simulations could help how students understand patient care. It’s about seeing healthcare as a collaborative effort, where clear communication and teamwork are essential.”—P2* *“Talking to a doctor, then they just want the important information in short, sharp bursts. […] one of my roles was to help educate the nurses […] you would talk to them in a slightly different way, and then the patients are going to be completely different again, so from an interdisciplinary point of view—computer simulation gives the students a bit of an idea of what to expect in hospital environment.”—P6*
Solution 2—role of champions	
Role of champions Number of participants = 6 Theme frequency = 7 Linked to barrier group: “educators’ resistance to change”	*“I think CBS really needs an advocate. […] you are not going to pay someone to convince you to use something unless you want to use it.”—P4* *“The role of a CBS champion is crucial in the educational landscape. […] advocating for the use of simulations […] providing a clear, actionable plan on how to integrate these tools seamlessly into the curriculum.”—P5* *“Having a champion to showcase the direct benefits of CBS can help overcome resistance and integrate these tools into the curriculum effectively.”—P9*
Solution 3—decrease educators’ workload	
Dedicated simulation technician Number of participants = 9 Theme frequency = 13 Linked to barrier group: “implementation barriers*—*(educators’ workload and time constraints)”	*“If we had a dedicated Educators member who could draft the cases for us. […] If we can say, ‘We want a case on this,’ and it could be written, then I think there is more chance that Educators will use it.”—P1* *“If there was a dedicated person preferably a pharmacist to make sure that it legally meets requirements and those kinds of things who had the time to take on all of the work, and I just handed them a case and said, ‘Here you go, what do you think? Do you think that there’s aspects of this that you could put into a simulation,’ and it was not on my shoulders, then absolutely yes.”—P8* *“Coming from, a pharmacist background, as I said, we know the dialogue. […] the reaction of the avatar, I found that part really difficult to program in. so, yeah, if that’s all done for you and you just have to make the questions relevant, that would be—that would be a big timesaver.”—P10*

CBS, computer-based simulation.

#### 3.2.1 Theme 1: resistance to change

One of the most significant barriers to CBS uptake identified in the interviews was resistance to change, which manifested in two primary subthemes: educator resistance and student resistance.

Educators expressed a preference for traditional teaching methods and scepticism towards CBS. This reluctance to embrace new technologies reflects a broader cultural resistance within the educational environment, where innovation is often met with caution and the need for extensive testing before adoption. Additionally, some educators view CBS as lacking educational merit and perceive them as games rather than legitimate educational tools.

In addition to educator resistance, the interviews revealed concerns about student resistance, particularly among international students. Some educators noted that these students tended to view CBS as less formal and less valuable compared to traditional methods. This highlights the importance of considering cultural perceptions and learning preferences when introducing new educational tools. Students who are accustomed to more conventional forms of instruction may find CBS challenging to accept, perceiving it as a departure from the rigour and structure they associate with effective learning.

#### 3.2.2 Theme 2: implementation and operational challenges

The second major theme was the implementation and operational challenges associated with integrating CBS into the curriculum. This theme encompasses several subthemes, including workload and time constraints and resource limitations.

Educators cited significant time constraints and increased workload required for developing case scenarios and adapting to CBS use. Balancing the initial setup of CBS with existing educational duties was highlighted as a major challenge, as it was seen as labour-intensive, involving the creation of complex case scenarios, testing, and refinement. Some educators expressed concerns that these demands could detract from other important responsibilities, making CBS a daunting addition to their teaching duties. However, many acknowledged that once CBS is fully integrated, it could reduce the ongoing workload compared to traditional methods, such as standardised patients and paper-based solutions, which need to be re-implemented annually. Despite recognising the potential long-term benefits of CBS, many felt that the upfront investment in time and resources could be a significant deterrent, particularly during the early stages of adoption. Another significant subtheme was the lack of adequate resources to support CBS implementation. Educators cited issues such as underpowered or malfunctioning computers, compounded by logistical challenges when institutions have multiple sites or campuses.

#### 3.2.3 Theme 3: CBS alignment with the curriculum

This theme includes subthemes such as curriculum integration and pedagogical effectiveness.

Some educators questioned the alignment of CBS with the existing pharmacy curriculum and its effectiveness in achieving learning objectives. They highlighted perceived gaps between the potential of CBS as an engaging tool and its actual effectiveness in delivering educational outcomes. Concerns were raised about whether CBS, in its current form, could be seamlessly integrated into the curriculum without compromising the quality of education. Educators were particularly worried that CBS might disrupt the flow of the curriculum and whether it could effectively complement other teaching methods. Additionally, design limitations were noted, such as difficulties in replicating real-life natural communication and a lack of customisation in CBS, which some educators felt could hinder the development of critical thinking and problem-solving skills.

### 3.3 Proposed solutions

Participants proposed several solutions that may facilitate overcoming these challenges. They emphasised the importance of communicating the benefits of CBS to stakeholders, fostering interprofessional learning opportunities through simulation, leveraging champions to advocate for technology integration, and alleviating educators’ workload by employing dedicated simulation technicians. Specifically, the solutions included promoting the benefits of CBS in complementing clinical placement training (18 instances, 10 participants) and employing dedicated CBS support staff, such as specialist simulation technicians (13 instances, 9 participants), to ease educator workloads.

#### 3.3.1 Solution 1: communicating CBS benefits

One of the proposed solutions focused on communicating the benefits of CBS more effectively, particularly in how CBS can complement existing training methods and foster interprofessional learning (IPL).

Educators suggested that CBS could play a critical role in complementing clinical placements, particularly by providing students with opportunities to practice and refine their skills in a controlled environment before interacting with real patients. This would help bridge the gap between theoretical knowledge and practical application, giving students confidence in their abilities. Additionally, CBS could offer real-time feedback, allowing students to learn from their mistakes in a safe setting. This approach would be especially valuable when traditional placements are limited or challenging to coordinate, as CBS can simulate scenarios that students might not otherwise encounter. The ability of CBS to supplement clinical placements was seen as a key factor in aligning it more closely with the pharmacy curriculum.

Another solution proposed was the use of CBS to enhance interprofessional training. By simulating real-life healthcare scenarios that involve multiple disciplines, CBS can help students understand the importance of communication and collaboration in patient care. Educators noted that IPL through CBS could overcome the logistical challenges of coordinating in-person IPL activities across different disciplines, making it a practical and effective tool for developing these essential skills. This solution directly addresses the subtheme of CBS alignment within the curriculum by integrating CBS as a means to achieve broader educational objectives beyond the scope of traditional, discipline-specific training.

#### 3.3.2 Solution 2: role of champions

To address resistance to change, educators proposed introducing champions—key individuals who advocate for CBS and guide its integration. Champions are seen as crucial in addressing the educators’ resistance to change. By demonstrating the benefits of CBS and offering practical support, champions can help shift the cultural attitudes within the educational environment. They would lead by example, showcasing successful CBS implementations and providing mentorship to other educators who are hesitant to adopt the technology. Champions could also play a pivotal role in aligning CBS with educational goals, ensuring that its integration is seamless and effective. This approach would help build confidence in CBS and foster a more supportive environment for its adoption.

#### 3.3.3 Solution 3: decreasing educators’ workload

A final proposed solution involves reducing the workload associated with CBS by employing dedicated simulation technicians. Educators suggested that this role could alleviate their burden by managing the technical aspects of CBS, such as scenario development, system maintenance, and troubleshooting. This approach would allow educators to concentrate on the pedagogical integration of CBS without being overwhelmed by operational demands. Having a team member who understands both the technical and educational aspects of CBS would also ensure its effective use, enhancing its value as a teaching tool. This solution addresses barriers related to educators’ workload and time constraints, making CBS a more viable option for curriculum integration.

## 4 Discussion

This case study explored the perceptions of students and educators towards using CBS in pharmacy education, uncovering several barriers impacting CBS uptake. These barriers encompassed cultural challenges, such as educators’ resistance to change, and operational challenges, including limited resources and substantial workload and time constraints faced by educators. Participants proposed several solutions to mitigate these barriers and enhance the uptake of CBS.

The combined insights from students and educators revealed both challenges and benefits to the broader adoption of CBS. There was strong student support for CBS, with 70.7% affirming its value for assessing knowledge and 80% endorsing its assistive role in education. These findings align with other educational contexts where technological readiness and positive perceptions towards digital learning tools have been increasingly noted. Studies have shown that digital natives, such as the current generation of students, are generally more receptive and adaptive to using technology for learning purposes (21).

However, only 16% of students believed that strong educator support for CBS integration was likely. The interviews with educators largely supported this view, exhibiting a notable resistance to change, potentially rooted in a preference for traditional teaching methods and concerns over the educational efficacy of new technologies. This resistance is compounded by practical barriers such as resource constraints and workload, hindering the effective deployment and integration of CBS into the curriculum. This phenomenon is well-documented in educational research, which underscores a natural hesitancy towards change (22–24). Introducing CBS requires an upfront investment and may be perceived as burdensome rather than enriching. Without adequate support, this workload can lead to burnout and resistance, despite the potential benefits for learning outcomes and longer-term workload reductions. Previous research has found that while students quickly adapt to new learning technologies, educators often lag due to factors such as resistance to change, lack of training, and perceived increase in workload (25).

This divergence in perspectives on the role of CBS in pharmacy education highlights a cultural gap that needs bridging. Overcoming faculty resistance and boosting institutional support are likely required to realise the full potential of CBS in pharmacy education. Students perceived CBS as highly valuable for improving patient communication, problem-solving abilities, and dispensing procedures. In contrast, educators expressed reservations about CBS’s ability to replicate complex real-world scenarios effectively and its alignment with educational outcomes. This limited active use suggests that their practical experience might not fully reflect CBS capabilities. Their feedback should be contextualised within their hands-on experience, highlighting the need for ongoing training and support to help educators integrate and utilise CBS effectively. The literature supports the positive views of students and refutes educators’ scepticism, with evidence that CBS can improve skills and knowledge, including clinical reasoning, procedural skills, and team skills (26). By integrating CBS more deeply into the curriculum, educational institutions can better prepare students for real-world clinical scenarios, enhancing their readiness for professional practice. Furthermore, the effective use of CBS can help bridge the gap between theoretical knowledge and practical application.

All interviewees supported CBS to complement placement training, recognising its value in addressing educational gaps. This support is reinforced by professional organisations such as the American Association of Colleges of Pharmacy (AACP) and the Accreditation Council for Pharmacy Education (ACPE), which advocate for using technologies like CBS as complementary teaching tools (27). CBS is also seen as having the potential to foster collaborative learning experiences and promote a culture of (IPL) within pharmacy education (28).

Our study revealed significant shortcomings in local institutional support, with nearly 40% of students questioning its adequacy. This underscores a critical gap in perceived support systems within the institution. Educators reported substantial challenges, notably heavy workloads and limited time, which hindered their ability to explore and adopt innovative teaching methods like CBS. To fully leverage the potential of CBS, schools must invest in infrastructure and provide ongoing training for educators. Such investments not only address current resistance but also empower educators with the skills and confidence needed to innovate in their teaching practices. As educators become more proficient with CBS, they can enhance the school’s overall development by creating more engaging and effective learning environments, which, in turn, can attract and retain students.

The literature consistently underscores the essential role of institutional support in successfully integrating new technologies in educational settings (29). One key suggested intervention is appointing a dedicated technician for CBS integration. This technician would provide essential technical support, facilitate smooth implementation, and allow educators to focus on pedagogical innovation rather than technical matters. By doing so, educators believed that educational institutions could ensure, CBS content is expertly managed and updated, enhancing both the quality and functionality of the simulations. Other targeted support measures identified in the literature may help address implementation challenges effectively (29). These include specialised training sessions for educators to grasp new technologies quickly without overwhelming them. Practical resources like customisable CBS templates and digital resource libraries can further streamline the time educators spend creating new content and integrating it into existing courses.

## 5 Study implications

Our study highlights the need for a strategic approach to equip both students and educators to engage effectively with CBS in pharmacy education.

For students, while their enthusiasm for CBS is evident, it is crucial to ensure they are not merely passive users but are actively developing the critical thinking and problem-solving skills that CBS can enhance. This requires carefully designed instructional strategies that integrate CBS into the curriculum in ways that align with professional practice expectations.

For educators, the study underscores the necessity of targeted faculty development initiatives. These should go beyond technical training to include pedagogical strategies for seamlessly incorporating CBS into teaching. The goal is to shift the perception of CBS from being an additional burden to a valuable tool that enhances educational outcomes. This shift can be facilitated by creating platforms for educators to share their experiences and successes with CBS, fostering a community of practice that supports continuous learning and adaptation.

Furthermore, the study suggests that institutional support plays a pivotal role in the successful adoption of CBS. This support includes not only providing the necessary infrastructure but also ensuring that educators have access to ongoing professional development and technical support. Such support structures are essential to overcoming the cultural and operational barriers identified in our study, enabling educators to innovate with confidence and effectiveness.

By addressing these implications, educational institutions can better prepare both students and educators to leverage CBS fully, ultimately enhancing the quality and effectiveness of pharmacy education. This will equip future pharmacists with the skills and knowledge needed to excel in an increasingly complex and technology-driven healthcare environment.

## 6 Limitations

The mixed-methods approach, combining qualitative interviews and quantitative surveys for two key stakeholders, ensured a comprehensive understanding of CBS uptake at UTAS and enhanced the reliability of the findings. Furthermore, the high response rate and detailed thematic analysis further strengthen the study’s credibility and relevance. However, the study sample, drawn from a single institution, may limit the generalisability of the findings. Nevertheless, the results are applicable in a broader context. The identified barriers and proposed solutions, such as dedicated support personnel and fostering interprofessional learning, address common issues in pharmacy education globally. These insights into overcoming challenges contribute to the wider adoption of CBS, benefiting pharmacy programs worldwide.

Additionally, the inclusion of students from different academic years may introduce bias due to uneven representation and varying levels of experience and familiarity with CBS. Moreover, there is a risk that participants might have provided socially desirable responses rather than their true perceptions, as the reliance on self-reported data from interviews and surveys may be subject to response bias. To mitigate this, we ensured the anonymity of responses, designed questions to maintain anonymity, and guaranteed that participation had no impact on students’ grades or study progress.

## 7 Recommendations for future research

Future research should incorporate larger, more diverse samples from multiple universities. This approach would enhance the accuracy and generalisability of the findings, providing a more comprehensive understanding of the barriers and facilitators to CBS adoption in pharmacy education. Additionally, gaining deeper insights into the perspectives of decision-makers would be useful for identifying potential barriers and developing strategies to overcome them.

## 8 Conclusion

This mixed-methods case study reveals a complex interplay of factors influencing the adoption of CBS in pharmacy education at our university, highlighting student enthusiasm and educator reservations. Despite students valuing the adoption of CBS, educators expressed concerns over practical implementation challenges, including infrastructure needs, workloads, and the impact on traditional teaching methods. Proposed solutions emphasised the need for dedicated support personnel, targeted training, and strategic curriculum integration.

## Data Availability

The original contributions presented in the study are included in the article/Supplementary material, further inquiries can be directed to the corresponding author.
