# Supplementary material for: Exploring barriers to the effective use of computer-based simulation in pharmacy education: a mixed-methods case study
Source: Front Med (Lausanne). 2024 Aug 29;11:1448893. doi: 10.3389/fmed.2024.1448893 (PMC11390378; doi:10.3389/fmed.2024.1448893)
Supplement: Supplementary file 1 [file Data_Sheet_1.PDF]

Supplementary Material

Supplementary Tables

Supplementary Table 1. Demographics of the survey participants (N=75)

**Student Category**	**Description**	**Number of Participants**<br><br>**(Total Participants Number = 75)**	**Percentage**
**Senior Students**	1 year remaining in their study	22	29.3%
**Mid-senior Students**	2 years remaining in their study	31	41.3%
**Junior Students**	3 years remaining in their study	22	29.3%

Supplementary Table 2. Student Perspectives on Resource Availability and CBS Integration in Pharmacy Curriculum (N=75)

Perspectives	Description	Statement	SA/A N (%)	Neither agree nor disagree N (%)	SD/D N (%)
**Resources Availability**	Considering the institutional infrastructure to support the use of CBS	Institution provides adequate access to computers	51 (68%)	8 (10.7%)	16 (21.3%)
		Institution provides a reliable internet connection	47 (62.7%)	6 (8%)	22 (29.3%)
		Institution support innovation in teaching (using technology in education)	31 (41.3%)	24 (32%)	20 (26.7%)
	Considering students ability to use CBS to study at home	Access to a suitable personal device at home	72 (96%)	1 (1.3%)	2 (2.7%)
		Reliable personal internet connection at home	71 (94.7%)	1 (1.3%)	3 (4%)
**CBS Role in Curricular Integration**	Considering personal preference for using CBS in students' learning	Assess my knowledge*	53 (70.7%)	13 (17.3%)	6 (8%)
		Use in the classroom#	47 (62.7%)	19 (25.3%)	8 (10.7%)
		Supplement classroom study	60 (80%)	7 (9.3%)	8 (10.7%)

*Abbreviations: A, agree; D, disagree; N, neutral; SA, strongly agree; SD, strongly disagree; *3 participants had no opinion; #1 participant had no opinion*

Supplementary Table 3. Demographics of the interview participants (N=10)

Educators Category	Characteristics
**Teaching Experience**	- 6 educators with over 5 years of experience<br>- 4 educators with 5 or fewer years of experience
**Roles**	Diverse roles spanning:<br>- Full-time and part-time positions as clinical instructors, lecturers, and tutors
**Experience using CBS**	- Only 2 out of the 10 interviewees actively using CBS in their teaching.<br>- 8 had previous exposure to CBS (trialled, or previously used it) but not actively using it now.

Appendix A – Students' Survey Questionnaire

Instructions: This survey may take up to 15 minutes to complete.

Section 1

A. Choose one of the following answers

1. How long is your pharmacy program at the University (full-time equivalent years)?
(Drop-down list with numbers 1-6)

2. How many years are left for you to finish/complete your university pharmacy program?
(Drop-down list with numbers 1-6)

3. Have you used computer-based simulation as part of your pharmacy practice or clinical pharmacy practice education? (Yes, No)

**If answered Yes – section B is required to be answered.*

B. Please select your answer from the drop-down lists

1. How often do you use a computer-based simulation tool to study outside scheduled school time?
(Drop down list of NEVER, SOMETIMES, OFTEN)

2. How often do you use a computer-based simulation tool in scheduled classes at your school?
(Drop down list of NEVER, SOMETIMES, OFTEN)

3. Do you think computer-based simulation is used often enough in your pharmacy practice/clinical pharmacy course/module?
(Drop down list of Not enough, the right amount, too often, Unable to judge)

Section 2

Using the categories below, choose the most appropriate response for each item:

2.1 When considering the computer-based simulation: Indicate how you rate the importance of each of the following elements *(very important=5 to not important at all=1).*

1. Realistic graphics
2. Ease of use
3. Bug-free experience
4. Availability of tech-support
5. Detailed feedback
6. Engaging content
7. Fun/enjoyable content

2.2 When considering the training focus for a computer-based simulation: Indicate how you rate the importance of each of the following elements (strongly agree=5 to strongly disagree=1).

1. Patient communication and counselling skills
2. Problem-solving skills
3. Dispensing procedure
4. Interprofessional communication skills
5. Hospital pharmacy practice
6. Community pharmacy practice
7. Community clinic practice (i.e. working in general practice/family doctor practice)

2.3 When considering your personal preference for using computer-based simulation. Indicate your level of agreement with each of the following statements (strongly agree=5 to strongly disagree=1).

1.	I would like to use a computer-based simulation tool to assess my knowledge
2.	I would like to use a computer-based simulation tool in the classroom
3.	I would like to use a computer-based simulation tool to supplement my classroom study

2.4 When considering your school infrastructure to support the use of computer-based simulation. Indicate your level of agreement with each of the following statements. (strongly agree=5 to strongly disagree=1)

1.	My school provides adequate access to computers
2.	My school provides a reliable internet connection
3.	My school embraces innovation in teaching

2.5 When considering your ability to use a computer-based simulation to study at home. Indicate your level of agreement with each of the following statements. (5=strongly agree to 1=disagree)

1.	I have access to a suitable personal device (i.e., PC, laptop, tablet, phone) at home
2.	I have a reliable personal internet connection at home
3.	I am comfortable using computer-based technology in my study

Section 3

Help: There will be questions regarding your perceptions and experience using one or more computer-based simulation tools.

3.1 How many computer-based simulation platforms have you used when studying Pharmacy practice/Clinical pharmacy?

3.2 What is the name of the tool you have used?

3.3 When did you last use this tool?

3.4 Based on your previous experience with the above-named computer-based simulation platform, indicate your level of agreement with each of the following statements (strongly agree=5 to strongly disagree=1).

Help: "Technical issues": an unexpected problem with the software such as (lag, bugs and glitches) that result in interfering with the program's performance.

1.	It has major technical issues that prevent you from completing the exercise
2.	It has minor technical issues that do not prevent you from completing the exercise
3.	Sufficient tutorials and online support on the simulator were provided
4.	It was easy to use
5.	It adequately replicates a real-world pharmacy practice experience
6.	It is an enjoyable way to study
7.	It is a time-efficient way to study
8.	It is an engaging way to study
9.	It should be used more in pharmacy practice education

3.5 If this simulation platform was missing an important feature, please, explain it below.

3.6 What did you like most about this platform?

3.7 What did you most dislike about this platform?

Section 4

4.1. Do you think pharmacy students would support the implementation of computer-based simulation in your pharmacy program?

Very likely	Somewhat likely	Not Likely

4.2. Can you please specify why did you choose this answer regarding the Students' stakeholder group?

4.3. Do you think educators would support the implementation of computer-based simulation in your pharmacy program?

Help: Educators: i.e. Teachers, lecturers...etc

Very likely	Somewhat likely	Not Likely

4.4. Can you please specify why did you choose this answer regarding the educators' stakeholder group?

4.5. Do you think leaders would support the implementation of computer-based simulation in your pharmacy program?

Help: leaders: i.e. Heads of schools, deans...etc

Very likely	Somewhat likely	Not Likely

4.6. Can you please specify why did you choose this answer regarding the leaders' stakeholder group?

4.7. Do you think any other barriers are preventing your school from implementing computer-based simulation which has not been covered by the previous questions?

4.8. Can you please discuss those barriers?

Appendix B - Educators' Interview Guide

I. Background Questions

- How long have you been assigned in your role?
  - ___________ years.
- Do you use computer-based simulation in your unit in pharmacy practice education?
  - Yes
  - No
- If answered "No", Are you aware of any available computer-based simulation in pharmacy practice training?
  - ___________
- If answered "Yes",
- How long have your unit been using the simulation tool?
  - ___________ years.
- Do you think computer-based simulation is used often enough in your unit/s?
  - ___________

(Not enough, the right amount, too often, Unable to judge)

II. Semi-structured Interview Topic Guide

Construct 1: General perceptions towards computer-based simulation

General Questions:

- What do you think of incorporating computer-based simulation in pharmacy practice education?

- Can you please, share your experience implementing computer-based simulation into your teaching unit(s)?

Construct 2: Questions about key stakeholders (School)

Questions for those who have experience

- Do you think your School would like to expand the use of computer-based simulations in your program?
- Do you think your school has adequate infrastructure (internet access, devices etc.) to support computer-based simulation?
- Can you please share your opinion regarding your pharmacy course - unit/s willingness to implement computer-based simulation?
- Do you think your Course – unit/s is well prepared to implement computer-based simulation?

Construct 3: Question about Key stakeholders (Educators)

General Questions:

- Do you feel educators in pharmacy education are adequately skilled to implement computer-based simulation within the curriculum?
- If an educator in your school wants to purchase or implement a new teaching tool for their class, what process would they have to go through?
- How does that relate to your willingness (and/or judgement) to incorporate computer-based simulation into your course - unit/s?
- In your opinion, how does the implementation of computer-based simulation, impact educators' workload or time?

Construct 4: Question about Key stakeholders (Students)

General Questions:

- In your opinion, to what extent do students at your course - unit/s support the implementation of computer-based simulation?
- Have you asked and/or involved students in the decision-making process regarding the use of computer-based simulation technology as a training tool?

Construct 5: Strategies for optimised implementation.

General Questions:

- In your opinion, what strategies or initiatives would help encourage the implementation of computer-based simulation?
- Are there certain assessment practices/frameworks you feel are effective to help your decision in implementing simulation training?
- In your opinion, what are the biggest challenges of implementing computer-based simulations?
- In your opinion, what are the most compelling reasons to implement computer-based simulations?

Construct 6: Any other comments you would like to share?

(Any additional comments, thoughts, or feedback would be much appreciated).
